# Supplementary material for: Carbonated mantle peridotites represent a hidden sink for subducted CO2
Source: Nat Commun. 2026 Feb 27;17:3297. doi: 10.1038/s41467-026-68646-3 (PMC13065980; doi:10.1038/s41467-026-68646-3)
Supplement: Supplementary file 1 — Supplementary Information [file 41467_2026_68646_MOESM1_ESM.pdf]

Supplementary text and figures for: Carbonated mantle peridotites represent a hidden sink for subducted CO_2

Elliot J. Carter[1,2]*, Brian O'Driscoll[3], Ray Burgess[1], Patricia L. Clay[3], Hélène Balcone-Boissard[4], Pierre Bürckel[5], and the Oman Drilling Project Science Team

[1]Department of Earth and Environmental Sciences, University of Manchester, Manchester, UK [2]School of Life Sciences, Keele University, Newcastle-under-Lyme, UK, [3]Department of Earth and Environmental Sciences, Ottawa University, Ottawa, Canada, [4]ISTeP, Sorbonne Université, CNRS, Paris, France, [5]Institut de Physique du Globe de Paris (IPGP), CNRS, Université de Paris, Paris, France

* Corresponding author: e.carter2@keele.ac.uk

1 Derivation of modelling equations

The following derivation demonstrates that the final halogen composition of a carbonating fluid depends upon the rates at which each halogen is lost from the rock during carbonation, the initial halogen ratios and the initial Cl/CO_2 ratio of the fluid. This can ultimately be expressed as a simple equation which relates the Cl/CO_2 of the fluid to the degree of halogen fractionation observed, fixing the relative abundance of Cl and CO_2 in the carbonating fluid and thereby helping to constrain its origin and associated carbon flux.

It should be noted that the least carbonated ophicarbonates have been affected by interaction with fractionated low Br/Cl fluids. A key assumption in the following calculations is therefore that compositional gradients of halogens and CO_2 content across the reaction zone (main text Figure 5) were in steady state, such that all samples analysed evolved (or would have evolved) along the same compositional gradient as a transient compositional front. If this is not the case, then the rate of Cl loss during carbonation may be overestimated relative to other halogens, resulting in underestimation of CO_2/Cl through the following calculations. That said, it is reasonable to think that such a steady state should have evolved since low Br/Cl fluids can only be maintained at low fluid/rock ratios, limiting their ability to supply excess Cl to uncarbonated rocks downstream. Conversely, increasing fluid/rock ratios increases the capacity of the fluids to deliver excess Cl downstream rocks but at the same time increases the Br/Cl of those fluids. These existence of low Br/Cl serpentinites affected by a fractionated fluid therefore suggests a balance between these countervailing tendencies. It is also possible that the least carbonated ophicarbonates are compositionally intermediate between their serpentinite protolith and the evolved

carbonating fluid, since they were most likely serpentinised prior to carbonation[1]. The effect of this would tend to cancel out any potential overestimation of Br/Cl_{out} and suggests that these assumptions and the modelling approach which follow are reasonable given the available constraints.

To begin with, we define the change in CO_2 per gram of rock or fluid:

$$\Delta[CO_2]_{rock} = \frac{CO_{2\,added}}{rock} \tag{1}$$

$$\Delta[CO_2]_{fluid} = \frac{CO_{2\,lost}}{fluid} \tag{2}$$

Where $\Delta[CO_2]_i$ is the change in the concentration of CO_2 in component i (in g/g), $CO_{2\,added}$ and $CO_{2\,lost}$ are the mass of CO_2 (in g) added to/lost from the rock or fluid, respectively, and rock and fluid are the mass (in g) of rock and fluid involved in the reaction.

Since the mass of CO_2 added to the rock is the same as that lost from the fluid

$$\Delta[CO_2]_{rock} \cdot rock = \Delta[CO_2]_{fluid} \cdot fluid \tag{3}$$

Rearranging (3) gives:

$$\frac{fluid}{rock} = \frac{\Delta[CO_2]_{rock}}{\Delta[CO_2]_{fluid}} \tag{4}$$

A minimum fluid/rock ratio can be calculated by assuming that all CO_2 from the fluid is consumed by the carbonation reaction:

$$\Delta[CO_2]_{fluid} = [CO_2]_{fluid} \tag{5}$$

$$\frac{fluid}{rock} = \frac{\Delta[CO_2]_{rock}}{[CO_2]_{fluid}} \tag{6}$$

To determine the amount of Cl, Br and I lost from the rock relative to the amount of CO_2 added during the reaction we define the ratios R_{Cl}, R_{Br} and R_I

$$R_{Cl} = \frac{\Delta[Cl]_{rock}}{\Delta[CO_2]_{rock}} \tag{7}$$

$$R_{Br} = \frac{\Delta[Br]_{rock}}{\Delta[CO_2]_{rock}} \tag{8}$$

$$R_I = \frac{\Delta[I]_{rock}}{\Delta[CO_2]_{rock}} \tag{9}$$

R_{Cl}, R_{Br} and R_I are determined by linear regression of bulk halogens and carbon abundance from reaction zone samples. Chlorine shows two distinct parts of the reaction, each with a different slope. There is rapid Cl loss during the early part of the

reaction (bulk CO_2 = 7 to 12%) followed by a shallower slope during the later part of the reaction (CO_2 = 12 to 40%). Alternatively, Cl loss can be fitted by exponential function as:

$$Cl = a \cdot e^{-kCO_2} \tag{10}$$

Where a is 0.1088 wt% and k = 0.12399 (with CO_2 in wt%). The rate of Cl loss against CO_2 is then itself dependent on CO_2 and given by:

$$R_{Cl} = \frac{dCl}{dCO_2} = -ka \cdot e^{-kCO_2} \tag{11}$$

$$R_{Cl} = -k \cdot Cl \tag{12}$$

Br and I by contrast appear to be lost more gradually throughout the reaction and are fit with a single linear slope. The result of this is that halogens are highly fractionated during the early part of the reaction, resulting in low Br/Cl and I/Cl ratios fluids expelled from the rock which mix with the carbon-bearing fluids.

The Br/Cl and I/Cl ratios of fluids expelled can be calculated by combing (7) or (10) with (8) and (9) as:

$$\left(\frac{Br}{Cl}\right)_{out} = \frac{R_{Br}}{R_{Cl}} \tag{13}$$

$$\left(\frac{I}{Cl}\right)_{out} = \frac{R_I}{R_{Cl}} \tag{14}$$

Calculated rates of halogen loss (as g halogen/g CO_2 added) are as follows:

R_{Cl} (g/g)	*Part 1*	-6.43×10^{-3}
	Part 2	-0.78×10^{-3}
	Exponential initial (CO_2 = 0)	-1.35×10^{-2}
	Exp. least carbonated (CO_2 = 7.1%)	-5.62×10^{-3}
R_{Br} (g/g)		-1.38×10^{-6}
R_I (g/g)		-0.10×10^{-6}

Since calculating an initial rate of Cl loss from the exponential model involves projecting beyond the sample data to zero CO_2, there is a risk this may not accurately reflect the behaviour of the system in the very earliest stage of the reaction (CO_2 = 0–7 wt%). Calculated at the CO_2 content of the least carbonated sample (CO_2 = 7.06 wt%), the exponential model gives a very similar R_{Cl} to part 1 of the two-stage linear model. Given this similarity and in the interests of simplicity, we use only the linear model hereafter. Combining R_{Cl} with R_{Br} and R_I from above gives the following halogen ratios for fluids lost from the rock during carbonation:

Br/Cl (g/g)	*Part 1*	0.21×10^{-3}
	Part 2	1.77×10^{-3}
I/Cl (g/g)	*Part 1*	16×10^{-6}
	Part 2	130×10^{-6}

To determine the effect of this outflux of halogens from the rock we need to calculate the composition of some mixture of this fluid and the original carbon-bearing fluid.

$$\left(\frac{Br}{Cl}\right)_f = \left(\frac{Br}{Cl}\right)_i \cdot X_{Cl} + \left(\frac{Br}{Cl}\right)_{out} \cdot (1 - X_{Cl}) \quad (15)$$

$$\left(\frac{I}{Cl}\right)_f = \left(\frac{I}{Cl}\right)_i \cdot X_{Cl} + \left(\frac{I}{Cl}\right)_{out} \cdot (1 - X_{Cl}) \quad (16)$$

Where Br/Cl and I/Cl are the weight ratio of the respective halogens, X_{Cl} is the weight proportion of Cl contributed by the original fluid and the subscripts *out*, *i* and *f* refer to the outflux from the rock, the initial fluid and the final fluid, respectively.

X_{Cl} can be expressed as:

$$X_{Cl} = \frac{[Cl]_i}{[Cl]_i + \Delta[Cl]_{fluid}} \quad (17)$$

Where [Cl]i is the initial Cl concentration in the fluid and $\Delta[Cl]_{fluid}$ is the change in concentration due Cl expelled from the rock.

To determine X_{Cl} in terms of the fluid Cl concentration and :

$$\Delta[Cl]_{fluid} = {\Delta[Cl]_{rock}} \Big/ \left(\frac{fluid}{rock}\right) \quad (18)$$

Where $\Delta[Cl]_i$ is the change in concentration of [Cl] (in g/g) in the respective component and

Substituting (6) in (18) (with the assumption of complete consumption of CO_2 from the fluid):

$$\Delta[Cl]_{fluid} = \Delta[Cl]_{rock} \cdot \frac{[CO_2]_{fluid}}{\Delta[CO_2]_{rock}} \quad (19)$$

Dividing by $[Cl]_i$ the initial fluid Cl concentration (in g/g) gives us:

$$\frac{\Delta[Cl]_{fluid}}{[Cl]_i} = \Delta[Cl]_{rock} \cdot \frac{[CO_2]_{fluid}}{\Delta[CO_2]_{rock}} \cdot \frac{1}{[Cl]_i} \quad (20)$$

Rearranging:

$$\frac{\Delta[Cl]_{fluid}}{[Cl]_i} = \frac{[CO_2]_{fluid}}{[Cl]_i} \cdot \frac{\Delta[Cl]_{rock}}{\Delta[CO_2]_{rock}} \tag{21}$$

Substituting definition of R_{Cl} (eq. (7) from above):

$$\frac{\Delta[Cl]_{fluid}}{[Cl]_i} = \frac{[CO_2]_{fluid}}{[Cl]_i} \cdot R_{Cl} \tag{22}$$

To reframe this equation in terms of X_{Cl} we can divide equation (17) by [Cl]i

$$X_{Cl} = \frac{1}{1 + \Delta[Cl]_{fluid}/[Cl]_i} \tag{23}$$

Substituting (22) into (23):

$$X_{Cl} = \frac{1}{1 + \frac{[CO_2]_{fluid}}{[Cl]_i} \cdot R_{Cl}} \tag{24}$$

We can now rearrange to calculate the initial CO_2/Cl ratio of the fluid from X_{Cl}.

$$X_{Cl}\left(1 + \frac{[CO_2]_{fluid}}{[Cl]_i} \cdot R_{Cl}\right) = 1 \tag{25}$$

$$\frac{[CO_2]_{fluid}}{[Cl]_i} \cdot R_{Cl} = \frac{1 - X_{Cl}}{X_{Cl}} \tag{26}$$

$$\frac{[CO_2]_{fluid}}{[Cl]_i} = \frac{1}{R_{Cl}}\left(\frac{1}{X_{Cl}} - 1\right) \tag{27}$$

Rearranging equation (15) as a level rule type relationship:

$$X_{Cl} = \frac{\left[\left(\frac{Br}{Cl}\right)_f - \left(\frac{Br}{Cl}\right)_{out}\right]}{\left[\left(\frac{Br}{Cl}\right)_i - \left(\frac{Br}{Cl}\right)_{out}\right]} \tag{28}$$

Finally combining equations (27) and (28):

$$\frac{[CO_2]_{fluid}}{[Cl]_i} = \frac{1}{R_{Cl}}\left(\frac{\left[\left(\frac{Br}{Cl}\right)_i - \left(\frac{Br}{Cl}\right)_{out}\right]}{\left[\left(\frac{Br}{Cl}\right)_f - \left(\frac{Br}{Cl}\right)_{out}\right]} - 1\right) \tag{29}$$

And equivalently:

$$\frac{[CO_2]_{fluid}}{[Cl]_i} = \frac{1}{R_{Cl}}\left(\frac{\left[\left(\frac{I}{Cl}\right)_i - \left(\frac{I}{Cl}\right)_{out}\right]}{\left[\left(\frac{I}{Cl}\right)_f - \left(\frac{I}{Cl}\right)_{out}\right]} - 1\right) \tag{30}$$

Equations (29) and (30) give the minimum initial CO_2/Cl ratio in the carbonating fluid needed to explain a given final fluid composition (Br/Cl_f or I/Cl_f). Alternatively, equation (24) can be used to calculate the relative proportions of Cl derived outflux from the rock versus the carbonating fluid. This can be input into equations (15) and (16) to calculate the composition of a fractionated fluid.

In either use case these numbers represent an endmember scenario, giving the minimum CO_2/Cl ratio required for a given degree of fractionation or the maximum fractionation for a given CO_2/Cl ratio. This is due to the assumption that all CO_2 present in the fluid is consumed by the reaction, implicitly fixing the fluid/rock ratio to its minimum possible value: that required to supply the amount of carbon present in the rock. In reality, the fluid CO_2 content should trend towards an equilibrium value. However, since XCO_2 of fluid in equilibrium with magnesite + talc + quartz is low (~0.0032)[2] it has been assumed that CO_2 is quantitatively removed from the fluid by the reaction. Fluid/rock ratios could be higher than the minimum bound calculated by this method; this would result in a lower degree of fractionation for a given CO_2/Cl ratio and conversely a higher CO_2/Cl ratio required to explain a given degree of fractionation.

Within our sample set we take the mean composition of serpentine in the least carbonated serpentinites 44z1 as representing the maximum degree of fractionation in the fluid. It is assumed that halogen ratios in serpentine reflect those of the fluid from which it formed, following Kendrick et al. (2013). The average composition of serpentine determined by SIMS in sample 44z1 is Br/Cl = $(1.07 \pm 0.57) \times 10^{-3}$ and I/Cl = $(1.01 \pm 0.55) \times 10^{-4}$ (n = 31).

2 Factors favouring forearcs peridotite carbonation

Forearc rocks and fluids may be sampled by dredging and drilling and in some cases, mud volcanism may sample material from the subduction channel at depth (≤20 km)[3,4]. Transit through accretionary prisms of sediment can modify the chemistry of fluids and so the characteristics of slab-derived volatiles and fluid-rock reactions are only easily assessed in non-accretionary subduction zones. The Marianas subduction zone is the type-example of a non-accretionary subduction zone[5] and is thus among the best-studied forearcs.

Carbonated peridotites have not been recovered from the Marianas or Tonga forearcs through dredging, nor sampled by mud volcanoes (e.g. ref. [3]). Carbonate is present in slab-derived fluids at depth >14 km[3,6,7] and results in precipitation of calcium carbonate in veins and voids but not bulk replacement of peridotite/serpentinite[6]

The lack of evidence for peridotite carbonation in the Marianas forearc may relate to differences in the thermal structure of the subduction zone and/or the supply of

sediment; Marianas is a cold subduction zone characterised by subduction of old crust (>150 Ma [8]) with low sediment carbonate contents (2.6% [9]). Thermodynamic modelling of decarbonation for the Marianas subduction zone suggest limited decarbonation (<0.5% [10]). In contrast, the exhumed metamorphic sole of the Oman Ophiolites shows evidence for high thermal gradients during subduction arising from the overlying hot young proto-ophiolite lithosphere, and resulting in plagiogranites derived from slab melting[11]. Warmer subduction regimes have been suggested to encourage devolatilisation of both water and carbon in the downgoing plate, resulting in preferential generation of carbon-rich aqueous fluids[12,13]. Migration of such fluids to shallower levels in the forearc without re-equilibration (e.g. via channelised flow along the slab interface) would be expected to result in significant carbonation.

Moreover, unlike those in the Mariana, subducting allochthonous Oman sediments were carbonate-rich with abundant reef carbonates and calc-siliciclastics in Haybi and Hawasina complexes, respectively[14,15]. The mean CO_2 content of the latter is 8.6%[15]. Thus, the composition of subducting sediment may too be an important factor in the generation of carbon-rich fluids and forearc listvenites. Relative to pure siliciclastic/volcanics or carbonates, mixed lithologies with 15-30% CO_2 have been suggested to result in the largest decarbonation fluxes due to the influence of aqueous fluids on carbonate stability[16]. It is therefore notable that along the Central American arc where there is perhaps the best evidence of ongoing carbonation at depth in the forearc[17] the subducting sediment is mixed siliciclastic-carbonate with ~25% CO_2 [9].

A further factor which has an important bearing on the formation of listvenites is the redox state of the fluid and the relative timing of serpentinisation and carbonation. Serpentinisation results in a reducing fluid and H_2 production and will encourage reduction of carbonate to methane[6]. As a result, listvenite formation is only likely to occur either under relatively oxidising conditions or after serpentinisation has completed. Textural relicts of serpentinite mesh texture within the BT1b listvenites strongly suggest that in Oman carbonation proceeded after serpentinisation[1].

Together these observations suggest that listvenite formation in forearcs may be encouraged by warm subduction, channelisation of deep fluids, subduction of carbonate-rich mixed siliciclastic sediment, oxidising conditions and/or prior serpentinisation of mantle wedge peridotites, although further work is necessary to clarify the relative importance of these factors. This raises the important possibility that the magnitude of the forearc carbon sink may vary spatially and temporally in response to differences in oceanic sedimentation, subduction thermal regime and redox state over geological time. Differences in sedimentation in particular are likely to have a strong influence on the CO_2 input to subduction and the redox state of fluids (e.g., [18,19]). Preferential forearc carbonation due to subduction of carbonate-bearing sediments may therefore have an important role in fluctuations in the long-term carbon cycle.

3 Supplementary figures – SIMS calibration and data quality

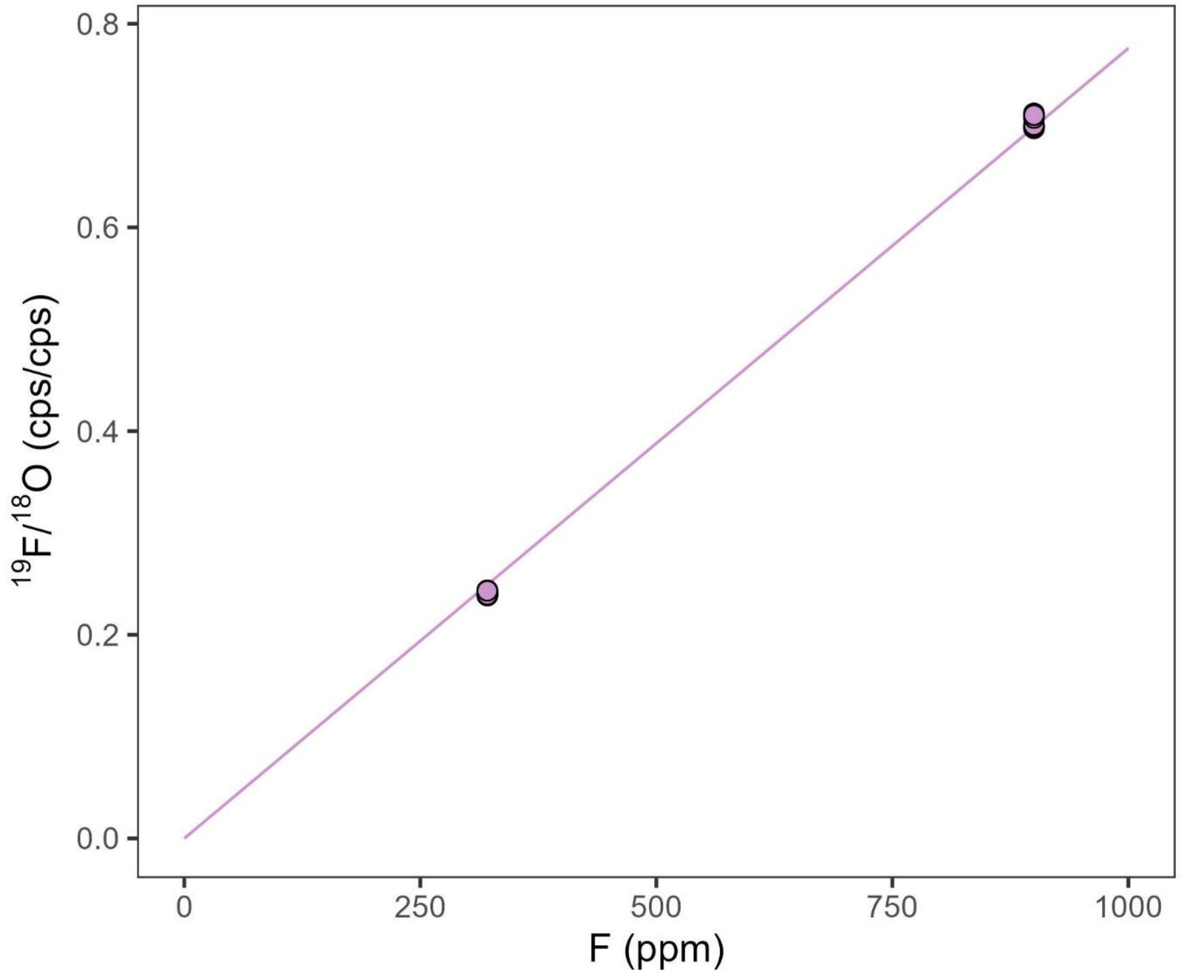

Supplementary Figure 1 – plot of SIMS $^{19}F/^{18}O$ signal ratio against certified F contents for two natural glass standards (T1G, ATHO) with widely differing matrices (SiO_2 = 50 and 75 wt%, respectively) and Cl contents (321, 900 ppm, respectively) showing linearity of SIMS response. SIMS standard data are given in Supplementary Data 9.

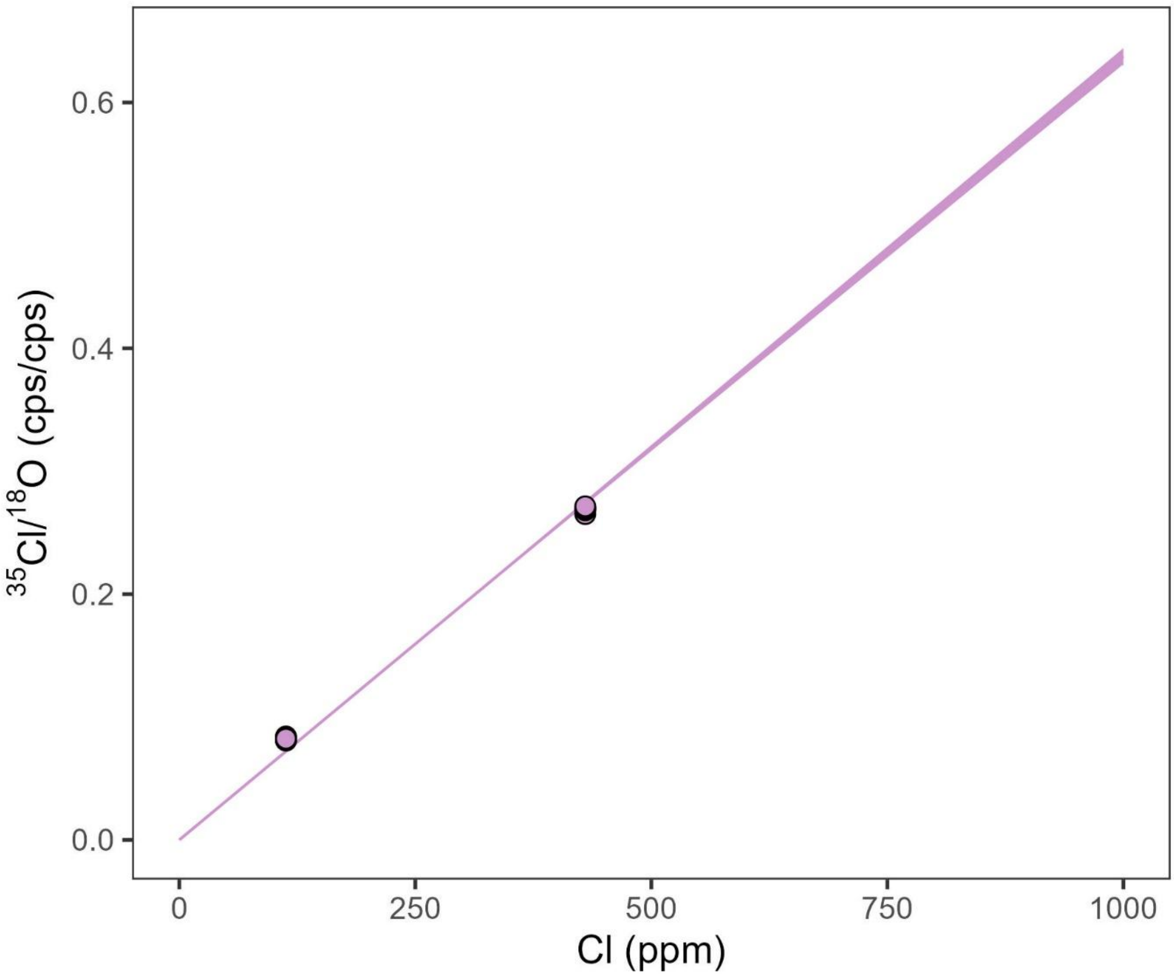

Supplementary Figure 2 – plot of SIMS $^{35}Cl/^{16}O$ signals against certified Cl contents for two natural glass standards (T1G, ATHO) with widely differing matrices (SiO_2 = 50 and 75 wt%, respectively) and Cl contents (100, 400 ppm, respectively) showing linearity of SIMS response and linear increase in uncertainty with Cl content. SIMS standard data are given in Supplementary Data 9.

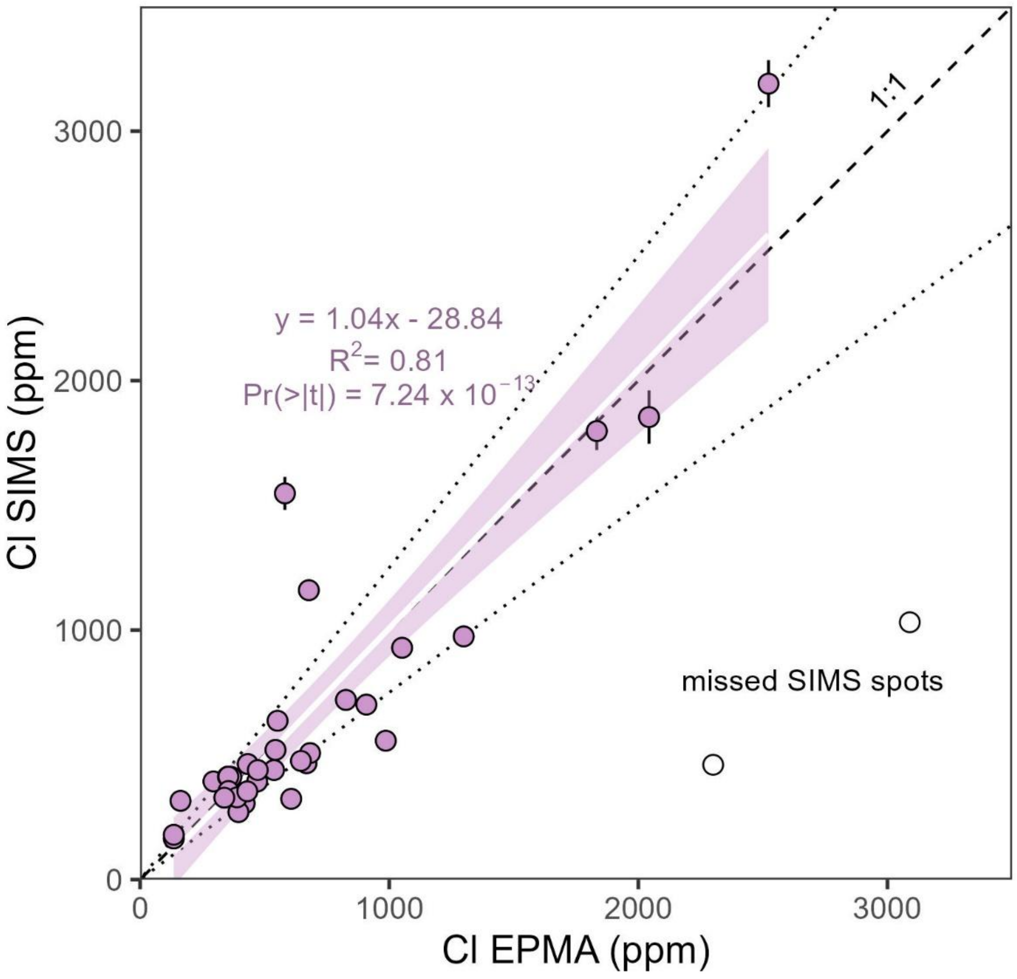

Supplementary Figure 3 – comparison of Cl data from EPMA and SIMS showing strong correlation (R^2 = 0.81) and linearity (m = 1.04). EPMA Cl data were derived from averaging of maps around spot locations as described in Methods. Two points with large excesses in Cl measured by EPMA relative to SIMS appear to be due to mistargeting of fine scale Cl-rich features visible on EPMA mapping but difficult to discern in reflected light.

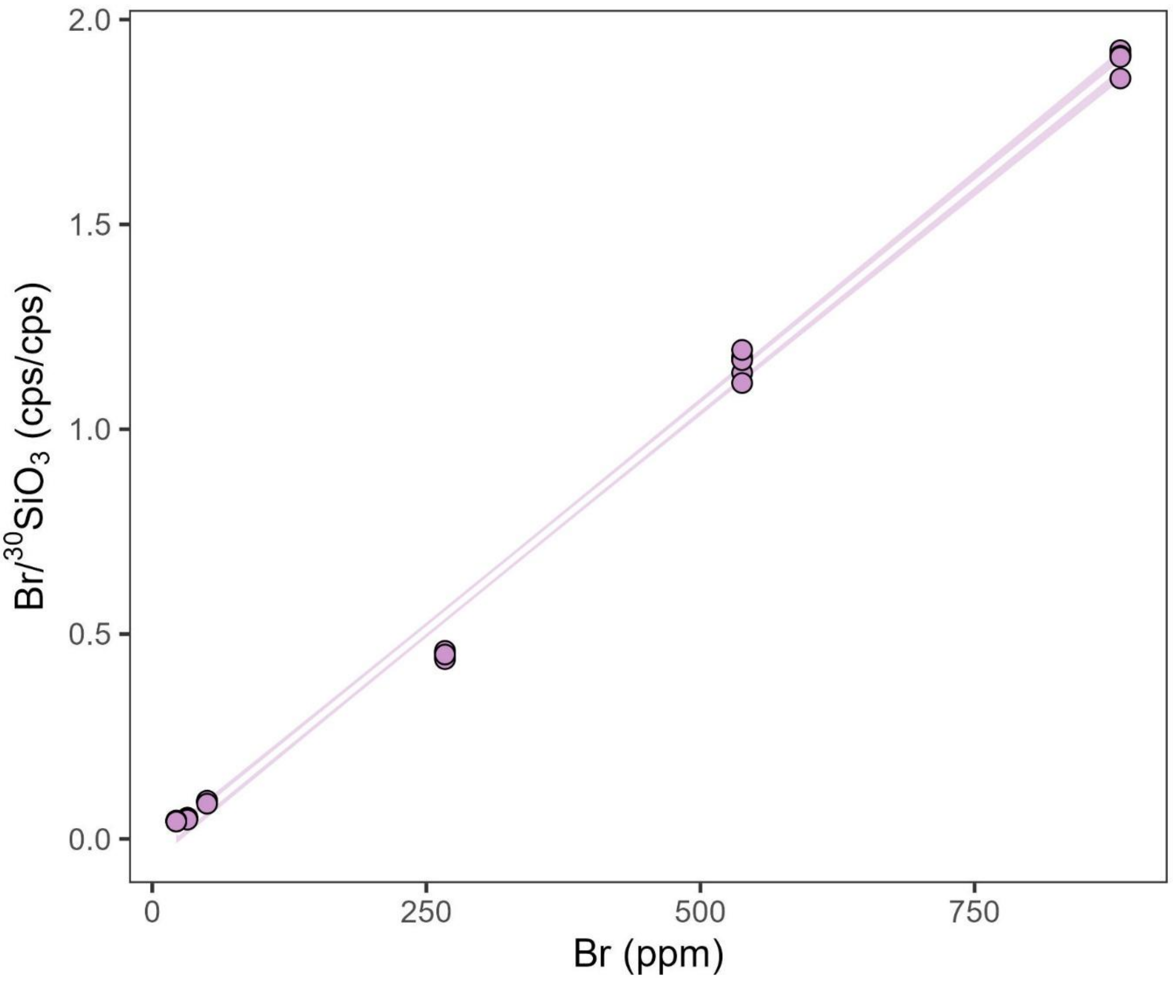

Supplementary Figure 4 – plot of SIMS Br/$^{30}Si^{16}O_3$ signal against Br content showing linearity across a range of standard matrices (scapolites BB1, BB2, SY, and synthetic glasses GSD and GSE) and Br contents (10-800 ppm). SIMS standard data are given in Supplementary Data 9.

References

1. Beinlich, A. *et al.* Ultramafic Rock Carbonation: Constraints From Listvenite Core BT1B, Oman Drilling Project. *J. Geophys. Res. Solid Earth* **125**, (2020).
2. Menzel, M. D., Sieber, M. J. & Godard, M. From peridotite to listvenite – perspectives on the processes, mechanisms and settings of ultramafic mineral carbonation to quartz-magnesite rocks. *Earth-Sci. Rev.* **255**, 104828 (2024).
3. Fryer, P., Wheat, C. G., Williams, T., & Expedition 366 Scientists. *Expedition 366 Summary*. vol. 366 (International Ocean Discovery Program, 2018).
4. Haggerty, J. A. Evidence from fluid seeps atop serpentine seamounts in the Mariana forearc: Clues for emplacement of the seamounts and their relationship to forearc tectonics. *Mar. Geol.* **102**, 293–309 (1991).
5. Noda, A. Forearc basins: Types, geometries, and relationships to subduction zone dynamics. *GSA Bull.* **128**, 879–895 (2016).
6. Albers, E. *et al.* Fluid-rock interactions in the shallow Mariana forearc: Carbon cycling and redox conditions. *Solid Earth* **10**, 907–930 (2019).
7. Mottl, M. J., Wheat, C. G., Fryer, P., Gharib, J. & Martin, J. B. Chemistry of springs across the Mariana forearc shows progressive devolatilization of the subducting plate. *Geochim. Cosmochim. Acta* **68**, 4915–4933 (2004).
8. Müller, R. D., Sdrolias, M., Gaina, C. & Roest, W. R. Age, spreading rates, and spreading asymmetry of the world's ocean crust. *Geochem. Geophys. Geosystems* **9**, (2008).
9. Plank, T. 4.17 - The Chemical Composition of Subducting Sediments. in *Treatise on Geochemistry (Second Edition)* (eds. Holland, H. D. & Turekian, K. K.) 607–629 (Elsevier, Oxford, 2014). doi:10.1016/B978-0-08-095975-7.00319-3.
10. Kerrick, D. M. & Connolly, J. a. D. Metamorphic devolatilization of subducted marine sediments and the transport of volatiles into the Earth's mantle. *Nature* **411**, 293–296 (2001).
11. Cox, J., Searle, M. & Pedersen, R. The petrogenesis of leucogranitic dykes intruding the northern Semail ophiolite, United Arab Emirates: field relationships, geochemistry and Sr/Nd isotope systematics. *Contrib. Mineral. Petrol.* **137**, 267–287 (1999).
12. Ribeiro, J. M. & Lee, C.-T. A. An imbalance in the deep water cycle at subduction zones: The potential importance of the fore-arc mantle. *Earth Planet. Sci. Lett.* **479**, 298–309 (2017).
13. Oyanagi, R. & Okamoto, A. Subducted carbon weakens the forearc mantle wedge in a warm subduction zone. *Nat. Commun.* **15**, 7159 (2024).

14. Searle, M. P. & Graham, G. M. “Oman Exotics”—Oceanic carbonate build-ups associated with the early stages of continental rifting. *Geology* **10**, 43–49 (1982).
15. de Obeso, J. C. *et al.* Deep Sourced Fluids for Peridotite Carbonation in the Shallow Mantle Wedge of a Fossil Subduction Zone: Sr and C Isotope Profiles of OmanDP Hole BT1B. *J. Geophys. Res. Solid Earth* **127**, 1–16 (2022).
16. Stewart, E. M. & Ague, J. J. Pervasive subduction zone devolatilization recycles CO2 into the forearc. *Nat. Commun.* **11**, 6220 (2020).
17. Barry, P. H. *et al.* Forearc carbon sink reduces long-term volatile recycling into the mantle. *Nature* **568**, 487–492 (2019).
18. Hu, W.-J. *et al.* The Redox State of Incipient Oceanic Subduction Zones: An Example From the Troodos Ophiolite (Cyprus). *J. Geophys. Res. Solid Earth* **128**, e2022JB025008 (2023).
19. Pälike, H. *et al.* A Cenozoic record of the equatorial Pacific carbonate compensation depth. *Nature* **488**, 609–614 (2012).
