# Supplementary material for: Carbonated mantle peridotites represent a hidden sink for subducted CO2
Source: Nat Commun. 2026 Feb 27;17:3297. doi: 10.1038/s41467-026-68646-3 (PMC13065980; doi:10.1038/s41467-026-68646-3)
Supplement: Supplementary file 2 — Description of Additional Supplementary Files [file 41467_2026_68646_MOESM2_ESM.pdf]

Description of Additional Supplementary Files

Supplementary Data 1 - Summary of preferred whole rock data for halogen, K and noble gas isotope abundances. F and F/Cl from pyrohydrolysis, all other values from neutron-irradiation noble gas mass spectrometry (NI-NG-MS). Full pyrohydrolysis and NI-NG-MS datasets available in Supplementary Data 2 and 2 respectively.

Supplementary Data 2 – Full dataset for whole rock halogen and sulphate abundances determined by pyrohydrolysis

Supplementary Data 3 – Full dataset and processing for whole rock halogens and heavy noble gas isotopes determined by neutron-irradiation noble gas mass spectrometry (NI-NG-MS)

Supplementary Data 4 – Summary of in situ halogen abundances determined by secondary ionisation mass spectrometry (SIMS) and electron probe microanalysis (EPMA)

Supplementary Data 5 – Full dataset for in situ halogens measured by secondary ionisation mass spectrometry (SIMS)

Supplementary Data 6 – Major elements, loss on ignition, and total carbon determined by X-ray fluorescence spectroscopy (XRF) and element analyser

Supplementary Data 7 - Bulk mineralogy determined by powder X-ray diffraction (XRD)

Supplementary Data 8 - Fluid evolution and CO_2 flux modelling calculations

Supplementary Data 9 - Secondary ionisation mass spectrometry (SIMS) data for glass and scapolite standards
